# The Causal Effect of Social Isolation on Cannabis Use Disorder and the Mediating Role of Depression: Evidence From a Mendelian Randomization Study

**DOI:** 10.1002/brb3.71102

**Published:** 2025-11-26

**Authors:** Tao Ma

**Affiliations:** ^1^ Investigation Department Hunan Police Academy Changsha China

**Keywords:** cannabis use disorder, causal inference, Mendelian randomization, social isolation

## Abstract

**Background:**

Observational studies suggest an association between social isolation and cannabis use disorder (CUD), but causality remains unclear. This study employs Mendelian randomization (MR) to assess the potential causal effect of social isolation on CUD and explore the mediating role of anxiety disorders and depression.

**Methods:**

GWAS summary statistics for social isolation, CUD, anxiety disorders, and depression were obtained from public GWAS repositories. Inverse variance weighted (IVW) was the primary MR method, supplemented by MR‐Egger, weighted median, and maximum likelihood approaches to evaluate: (i) The causal effect of social isolation on CUD; (ii) its effect on anxiety disorders and depression; (iii) the effect of anxiety disorders and depression on CUD. Sensitivity analyses included Cochran's Q test for heterogeneity, MR‐Egger intercept and MR‐PRESSO for pleiotropy, and leave‐one‐out analysis for robustness. The mediation effect was quantified using the delta method.

**Results:**

IVW analysis revealed a significant positive correlation between social isolation and increased CUD risk (OR = 4.29, 95% CI: 1.35–13.64, *p* = 0.014), with supplementary MR methods yielding consistent results (OR > 1). Sensitivity analyses confirmed the robustness of findings. In addition, the mediation MR analysis revealed that depression significantly mediated the causal effect of social isolation on CUD. Specifically, social isolation showed a significant positive association with depression risk (OR = 3.70, 95% CI: 2.32–5.89, *p* = 3.67E‐08), and depression, in turn, was positively associated with an increased risk of CUD (OR = 1.27, 95% CI: 1.08–1.50, *p* = 0.003). The delta method indicated that depression mediated 21.8% of the effect of social isolation on CUD risk.

**Conclusions:**

Social isolation is potentially associated with an increased risk of CUD, with depression as a key mediator. The findings should be considered in light of limitations including potential recall bias, European ancestry samples, and the inability to assess exposure‐mediator interactions using summary‐level data.

## Graphical abstract

This study utilized large‐scale GWAS summary statistics to conduct a two‐sample Mendelian randomization (MR) analysis, revealing that social isolation significantly increases the risk of cannabis use disorder. Furthermore, mediation MR analysis indicated that depression mediates 21.8% of the causal effect.

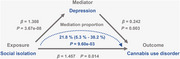

## 1 Introduction

Cannabis is among the most widely used psychoactive substances worldwide as legalization expands across various regions (Ransing et al. 2022). Despite its increasing social acceptance, excessive and prolonged cannabis use can lead to cannabis use disorder (CUD), a psychiatric condition characterized by compulsive cannabis consumption, impaired control over use, and significant distress or functional impairment (Connor et al. 2021). CUD poses severe health risks, including a heightened likelihood of cardiovascular diseases such as atrial fibrillation, heart failure, and stroke, as well as respiratory complications like chronic bronchitis and impaired lung function (M. Chen et al. 2022; Gracie and Hancox 2021). Given these substantial health risks, it is crucial to understand the underlying factors contributing to CUD development to improve prevention and intervention strategies.

Beyond individual behavioral patterns, broader psychosocial and environmental factors play a crucial role in shaping cannabis use behaviors and CUD risk (Beck et al. 2009). Among these, social isolation has gained increasing attention as a potential determinant of substance use. Social isolation is defined as a prolonged lack of social contact and meaningful interactions, often characterized by minimal engagement with family, friends, or community networks, as well as reduced participation in social activities (Cacioppo and Hawkley 2003). Epidemiological evidence suggests that social isolation and loneliness are highly prevalent, with over 25% of U.S. adults living alone and more than one‐third of individuals over 45 experiencing persistent social disconnection (Holt‐Lunstad 2017). The COVID‐19 pandemic further exacerbated this issue, with increased isolation linked to higher rates of cannabis use (Bartel et al. 2020).

However, most studies examining the association between social isolation and CUD are observational, limiting their ability to establish causal relationships. To address this limitation, this study employs Mendelian randomization (MR) analysis, a genetic epidemiological approach that leverages genetic variants as instrumental variables (IVs) to strengthen causal inference (Sanderson et al. 2022). By exploiting the natural random allocation of genetic variants during meiosis, MR simulates a randomized controlled trial, effectively minimizing confounding and reverse causation (Swanson et al. 2017). In this study, single‐nucleotide polymorphisms (SNPs) are selected as IVs to proxy social isolation, and summary‐level data from genome‐wide association studies (GWAS) are used to estimate its causal effect on CUD.

This study used a two‐sample MR approach to investigate the causal relationship between social isolation (exposure) and CUD (outcome), addressing the limitations of observational studies and providing stronger evidence for causality. We further applied a mediation MR framework to assess whether anxiety disorders or depression mediated this association. As social isolation has been identified as a risk factor for anxiety disorders and depression, this analysis emphasized the importance of regular psychological support and mental health interventions for socially isolated individuals as a potential strategy to prevent CUD.

## 2 Materials and Methods

### 2.1 Study Design

This study aimed (1) to determine whether there is evidence supporting a potential causal association between social isolation and CUD, and (2) to evaluate whether anxiety or depression mediates this relationship. The analyses were conducted within a two‐sample and mediation MR framework. In brief, the study was carried out in the following steps: (1) Retrieving GWAS summary statistics for social isolation, CUD, anxiety, and depression from relevant GWAS repositories; (2) Assessing the causal effect of social isolation on CUD; (3) Evaluating the causal effect of social isolation on anxiety/depression; (4) Assessing the causal effect of anxiety/depression on CUD; (5) Calculating the proportion of mediation. All analyses were conducted using R software (version 4.3.1). The primary analyses were performed using the TwoSampleMR package (version 0.6.8), RadialMR (version 1.1), and MR‐PRESSO (version 1.0). The GWAS summary statistics used in this study were all obtained from public databases, and the corresponding original studies had received ethical approval. Therefore, no additional ethical approval or informed consent was required for this study. This study was conducted based on the STROBE‐MR guidelines (Skrivankova et al. 2021).

### 2.2 Definition of Traits and Obtaining of GWAS Summary Statistics

The summary statistics for exposure, mediator, and outcome GWAS in this study were all derived from non‐overlapping European cohorts. Detailed information and access links for all GWAS summary statistics used in this study are listed in Table S1. The exposure, mediator, and outcome GWAS were obtained from different cohorts, and any potential sample overlap was expected to be minimal.

#### 2.2.1 Social Isolation

The GWAS summary statistics for social isolation were obtained from the IEU OpenGWAS project database. This trait was assessed in the UK Biobank (UKB), a large population‐based cohort study collecting genetic, health, and lifestyle data from participants in the United Kingdom. Social isolation was measured using a computerized questionnaire, based on responses to the question: “Do you often feel lonely?” Individuals who answered “yes” were classified as cases (*n* = 58,752), while those who answered “no” were controls (*n* = 273,511). The dataset provides strong statistical power for identifying genetic factors linked to social isolation.

#### 2.2.2 Cannabis Use Disorder

The GWAS summary statistics for CUD were obtained from the Psychiatric Genomics Consortium (PGC) database. CUD is a psychiatric condition characterized by persistent cannabis use despite significant impairment or distress, meeting the diagnostic criteria of DSM‐IV, DSM‐III‐R, or DSM‐5 for cannabis abuse or dependence. The GWAS summary statistics were derived from a large‐scale GWAS meta‐analysis conducted by Johnson et al., which included 18 cohorts from the PGC—Substance Use Disorders working group, along with data from the iPSYCH (Danish cohort) and deCODE (Icelandic cohort) studies (Johnson et al. 2020). We specifically used data from European‐ancestry cohorts, including only unrelated individuals from genotyped samples (14,080 cases and 343,726 controls), excluding related individuals from the PGC family‐based samples.

#### 2.2.3 Anxiety Disorders/Depression

The GWAS summary statistics for anxiety disorders and depression were obtained from the FinnGen consortium database. Anxiety disorder is a psychiatric condition characterized by persistent feelings of anxiety or fear, often accompanied by physical symptoms. Depression is a mood disorder marked by prolonged episodes of psychological distress, loss of interest or pleasure, and impairments in appetite, sleep, energy, and decision‐making. This study included samples from FinnGen's latest R12 cohort, comprising 35,875 anxiety disorder cases and 444,414 controls and 59,333 depression cases and 434,831 controls.

### 2.3 Selection of Genetic IVs

The IVs (SNPs) used for MR analysis satisfy three core assumptions: (1) Relevance, i.e., the IVs are strongly associated with the exposure; (2) Independence, i.e., the IVs are not associated with potential confounders; (3) Exclusion restriction, i.e., the IVs influence the outcome only through the exposure. SNPs highly associated with the exposure were used as proxies for the exposure in MR analysis. First, SNPs were selected from the GWAS summary statistics based on a genome‐wide significance threshold of *P* < 5e‐8. To ensure the independence of IVs, SNPs in linkage disequilibrium (*r* ^2^ ≥ 0.001 within a 10,000 kb region) were excluded. If the number of independent IVs was insufficient (< 10), the significance threshold was relaxed to *p* < 5e‐6 to maintain adequate statistical power for the MR analysis. Next, palindromic SNPs were removed by setting the “action” parameter in the “harmonise_data” function to 3. In addition, the RadialMR R package was used to filter out outlier SNPs. Finally, the strength of the IVs was evaluated by calculating the Cragg‐Donald *F*‐statistic (Burgess and Thompson 2011). The *F*‐statistic for each IV was computed using the formula: *F*‐statistic = (*n* − 2) * *R* ^2^ / (1 − *R* ^2^), where *n* is the sample size and *R* ^2^ indicates the proportion of variance in the exposure explained by the IV. The *R* ^2^ value was obtained using the “add_rsq” function from the “TwoSampleMR” R package. To mitigate the risk of weak instrument bias, only IVs with an *F*‐statistic greater than 10 were included in the final MR analysis. In addition, the MR Steiger test was applied to assess the validity of IVs and to minimize the possibility of reverse causation.

### 2.4 Statistical Analysis

#### 2.4.1 Two‐Sample MR Analysis

For causal estimation, the inverse variance weighted (IVW) method served as the primary approach. Specifically, the Wald ratio method was employed to estimate the causal effect of exposure on an outcome when using a single IV. A meta‐analysis was then performed using a random‐effects model to derive the IVW causal estimate (Pagoni et al. 2019). In addition, several complementary MR methods, including MR‐Egger, weighted median, and maximum likelihood, were applied to enhance the robustness of the findings. The MR‐Egger method was particularly useful in detecting and adjusting for horizontal pleiotropy (Burgess and Thompson 2017). The weighted median method provided reliable estimates even when up to 50% of the IVs violated the core MR assumptions (Bowden et al. 2016). Meanwhile, the maximum likelihood approach, a traditional statistical technique that estimates distribution parameters by maximizing the likelihood function, yielded lower standard errors compared to other methods (Milligan 2003). Since the outcomes in this study were binary variables, the results of the MR analysis were expressed as odds ratios (OR) with 95% confidence intervals (CI). A causal relationship was considered statistically significant only if the *p*‐value of the IVW MR estimate was below 0.05, and the OR direction derived from at least three additional MR methods was consistent with the IVW estimate.

#### 2.4.2 Sensitivity Analysis

To ensure the reliability and robustness of the MR estimates, multiple sensitivity analyses were performed. Cochran's Q test was used to assess heterogeneity, specifically evaluating the variability in causal effect estimates across individual SNPs. Non‐significant heterogeneity suggests that the SNPs have consistent effects, supporting the reliability of MR assumptions. The MR‐Egger intercept test was applied to detect horizontal pleiotropy, where genetic variants influence the outcome through pathways independent of the exposure—a nonzero intercept suggests pleiotropy that could bias MR results. MR‐PRESSO (Mendelian Randomization Pleiotropy RESidual Sum and Outlier) global test was used to identify and correct for pleiotropic outlier SNPs, improving the robustness of the causal inference. In addition, leave‐one‐out analysis was conducted by sequentially removing each SNP and recalculating the MR estimates to assess whether a single SNP disproportionately influenced the results.

#### 2.4.3 Mediation MR Analysis

A two‐step mediation MR analysis was conducted to assess whether anxiety disorders or depression mediate the causal relationship between social isolation and CUD. Specifically, anxiety disorders or depression were considered potential mediators if social isolation was significantly associated with them, and they were, in turn, significantly associated with the risk of CUD. The mediation effect proportion and its corresponding 95% CI were estimated using the delta method (Carter et al. 2019). In this framework, *β*_1_ represents the estimated causal association of social isolation with the mediator (with a standard error of SE(*β*_1_)), *β*_2_ represents the causal association of the mediator with CUD (with a standard error of SE(*β*_2_)), and *β*_3_ represents the total association of social isolation with CUD (with a standard error of SE(*β*_3_)). The mediation proportion was calculated as (*β*_1_ × *β*_2_) / *β*_3_, with its 95% CI determined using the following formula: $\frac{\beta_{1}\times \beta_{2}\pm 1.96\times \sqrt{{\beta_{1}}^{2}\times {\left(\mathrm{SE}\left(\beta_{2}\right)\right)}^{2}+{\beta_{2}}^{2}\times {\left(\mathrm{SE}\left(\beta_{1}\right)\right)}^{2}}}{\beta_{3}}$.

## 3 Results

### 3.1 MR Analysis Supports a Significant Positive Causal Association Between Social Isolation and CUD

Table S2 presents in detail the IVs used for MR analysis to evaluate the causal relationship between social isolation and CUD. Thirty‐seven independent IVs proxying social isolation within the significance threshold were finally included, and all *F*‐statistics were > 10, suggesting strong IVs. All IVs passed the MR Steiger test.

**FIGURE 1:**
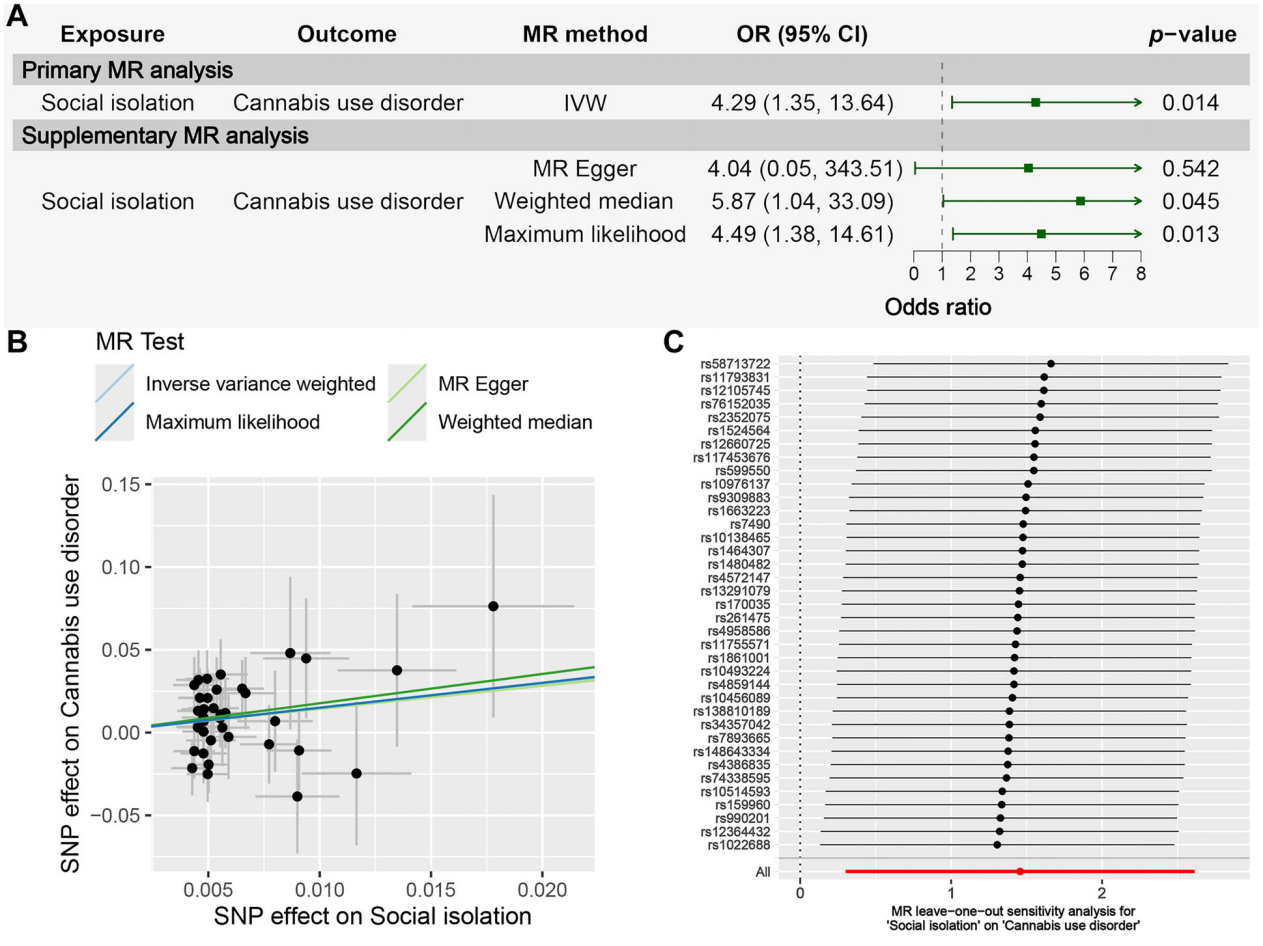
MR results for assessing the causal effect of social isolation on CUD. (A) Forest plot of the MR analysis assessing the causal effect of social isolation on CUD. (B) Scatter plot of the SNP effect on social isolation and CUD. (C) MR leave−one−out sensitivity analysis for social isolation on CUD.

Table 1 and Figure 1A present the MR results assessing the causal effect of social isolation on CUD. The primary MR method, IVW, demonstrated that increased social isolation was significantly associated with a higher likelihood of CUD (OR = 4.29, 95% CI: 1.35–13.64, *p* = 0.014). Three additional MR methods (MR‐Egger, weighted median, and maximum likelihood) produced consistent results (OR > 1), reinforcing the IVW findings. The scatter plot visually demonstrates the evidence from four MR methods supporting this causal association (Figure 1B).

**TABLE 1:** Causal effect of social isolation on CUD assessed by four MR methods.

Exposure	Outcome	MR method	OR (95% CI)	*p*‐value
Social isolation	Cannabis use disorder	IVW	4.29 (1.35, 13.64)	0.014
		MR Egger	4.04 (0.05, 343.51)	0.542
		Weighted median	5.87 (1.04, 33.09)	0.045
		Maximum likelihood	4.49 (1.38, 14.61)	0.013

Several sensitivity analyses were then performed to assess the reliability of the MR results (Table S3). First, the Cochrane's *Q*‐test test indicated that the MR analysis was not significantly affected by heterogeneity (*Q*_IVW_ = 30.37, *Q*_IVW__pval = 0.73 > 0.05; *Q*_Egger_ = 30.37, *Q*_Egger_ _pval = 0.69 > 0.05). Next, the MR‐Egger intercept test (intercept = 3.55E‐04, *p* = 0.98 > 0.05) and the MR‐PRESSO global test (RSSobs = 32.10, *p* = 0.74 > 0.05) suggested no significant horizontal pleiotropy. Finally, the leave‐one‐out analysis showed that replicated analyses after excluding any single IV still indicated a significant causal association, suggesting that no outlier SNPs influenced the overall results (Figure 1C). Overall, these sensitivity analyses further support the positive causal relationship between social isolation and CUD.

### 3.2 MR Analysis Indicated Depression Mediated the Causal Relationship Between Social Isolation and CUD

#### 3.2.1 Causal Effect of Social Isolation on Anxiety Disorders/Depression

Tables S4 and S5 provide detailed information on the IVs used in the MR analysis to assess the causal relationship between social isolation and anxiety disorders or depression. A total of 38 and 34 independent IVs, respectively, were selected within the significance threshold to evaluate the causal effect of social isolation on anxiety disorders and depression. All IVs exhibited *F*‐statistics > 10, confirming their robustness. All IVs passed the MR Steiger test.

**FIGURE 2:**
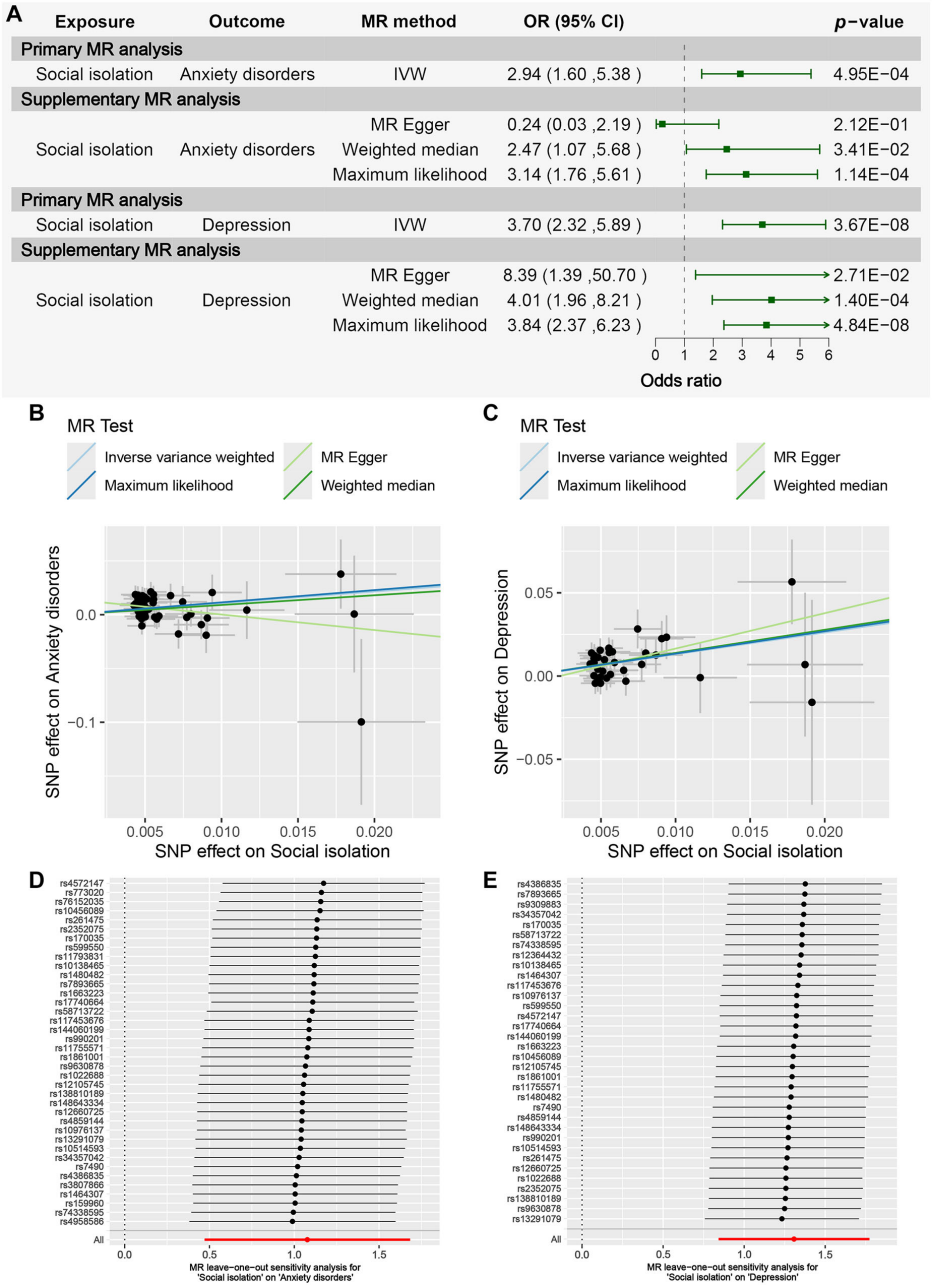
MR results for assessing the causal effect of social isolation on anxiety disorders/depression. (A) Forest plot of the MR analysis assessing the causal effect of social isolation on anxiety disorders/depression. (B) Scatter plot of the SNP effect on social isolation and anxiety disorders. (C) Scatter plot of the SNP effect on social isolation and depression. (D) MR leave−one−out sensitivity analysis for social isolation on anxiety disorders. (E) MR leave−one−out sensitivity analysis for social isolation on depression.

Table 2 and Figure 2A present the MR results evaluating the causal effect of social isolation on anxiety disorders and depression. The primary MR method, IVW, indicated that increased social isolation was significantly associated with a higher risk of anxiety disorders (OR = 2.94, 95% CI: 1.60–5.38, *p* = 4.95E‐04) and depression (OR = 3.70, 95% CI: 2.32–5.89, *p* = 3.67E‐08). Notably, the MR‐Egger method suggested an opposite effect (OR < 1) when assessing the causal relationship between social isolation and anxiety disorders. In contrast, three supplementary MR methods consistently supported a positive causal association between social isolation and depression (OR > 1). Figure 2B,C illustrate the scatter plots for social isolation versus anxiety disorders and depression, respectively.

**TABLE 2:** Causal effect of social isolation on anxiety disorders/depression assessed by four MR methods.

Exposure	Outcome	MR method	OR (95% CI)	*p*‐value
Social isolation	Anxiety disorders	IVW	2.94 (1.60, 5.38)	4.95E‐04
		MR Egger	0.24 (0.03, 2.19)	2.12E‐01
		Weighted median	2.47 (1.07, 5.68)	3.41E‐02
		Maximum likelihood	3.14 (1.76, 5.61)	1.14E‐04
Social isolation	Depression	IVW	3.70 (2.32, 5.89)	3.67E‐08
		MR Egger	8.39 (1.39, 50.70)	2.71E‐02
		Weighted median	4.01 (1.96, 8.21)	1.40E‐04
		Maximum likelihood	3.84 (2.37, 6.23)	4.84E‐08

Subsequently, sensitivity analyses were performed (Table S6). The Cochrane's *Q*‐test indicated no significant heterogeneity ([i] anxiety disorders: *Q*_IVW_ = 43.24, *Q*_IVW__pval = 0.22 > 0.05; *Q*_Egger_ = 37.72, *Q*_Egger_ _pval = 0.39 > 0.05; [ii] depression: *Q*_IVW_ = 31.87, *Q*_IVW__pval = 0.52 > 0.05; *Q*_Egger_ = 31.02, *Q*_Egger_ _pval = 0.52 > 0.05). According to the MR‐Egger intercept test and the MR‐PRESSO global test, horizontal pleiotropy was present in the analysis of anxiety disorders (MR‐Egger intercept test: Intercept = 0.01, *p* = 0.03 < 0.05; MR‐PRESSO global test: RSSobs = 45.62, *p* = 0.24 > 0.05) but not in that of depression (MR‐Egger intercept test: intercept = −4.77E‐03, *p* = 0.36 > 0.05; MR‐PRESSO global test: RSSobs = 33.88, *p* = 0.57 > 0.05). The leave‐one‐out analysis confirmed no outlier SNPs (Figures 2D,E). Overall, the above MR results and sensitivity results suggest that the causal effect of social isolation on depression is more significant and stable compared to anxiety disorders.

#### 3.2.2 Causal Effect of Anxiety Disorders/Depression on CUD

Tables S7 and S8 provide detailed information on the IVs used in the MR analysis to assess the causal relationship between anxiety disorders/depression and CUD. A total of 10 and 27 independent IVs were selected to proxy anxiety disorders and depression, respectively. Notably, all *F*‐statistics exceeded 10, indicating robust IVs. All IVs passed the MR Steiger test.

**FIGURE 3:**
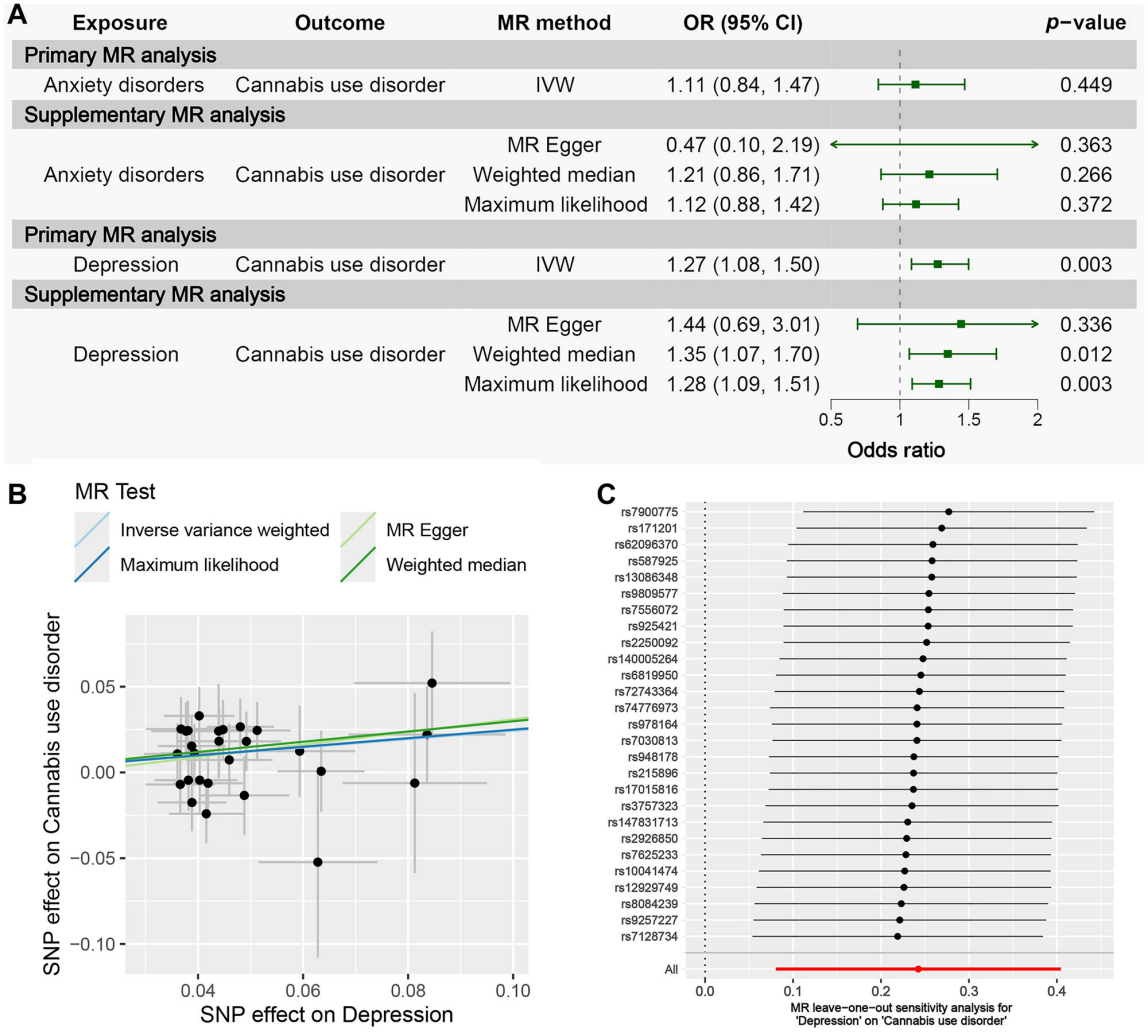
MR results for assessing the causal effect of anxiety disorders/depression on CUD. (A) Forest plot of the MR analysis assessing the causal effect of anxiety disorders/depression on CUD. (B) Scatter plot of the SNP effect on depression and CUD. (C) MR leave−one−out sensitivity analysis for depression on CUD.

Table 3 and Figure 3A present the MR results assessing the causal effect of anxiety disorders and depression on CUD. The IVW analysis found no evidence of a causal association between anxiety disorders and CUD (*p* > 0.05). In contrast, IVW indicated a significant causal relationship between genetic liability to depression and an increased risk of CUD (OR = 1.27, 95% CI: 1.08–1.50, *p* = 0.003). In addition, three supplementary MR methods yielded consistent results with IVW (OR > 1), further supporting this association. Figure 3B displays the scatter plot for depression versus CUD, visually illustrating their significant causal relationship.

**TABLE 3:** Causal effect of anxiety disorders/depression on CUD assessed by four MR methods.

Exposure	Outcome	MR method	OR (95% CI)	*p*‐value
Anxiety disorders	Cannabis use disorder	IVW	1.11 (0.84, 1.47)	0.449
		MR Egger	0.47 (0.10, 2.19)	0.363
		Weighted median	1.21 (0.86, 1.71)	0.266
		Maximum likelihood	1.12 (0.88, 1.42)	0.372
Depression	Cannabis use disorder	IVW	1.27 (1.08, 1.50)	0.003
		MR Egger	1.44 (0.69, 3.01)	0.336
		Weighted median	1.35 (1.07, 1.70)	0.012
		Maximum likelihood	1.28 (1.09, 1.51)	0.003

Subsequently, sensitivity analyses were conducted (Table S9), showing no evidence of significant heterogeneity (Cochrane's *Q*‐test: *Q*_IVW_ = 20.90, *Q*_IVW__pval = 0.75 > 0.05; *Q*_Egger_ = 20.79, *Q*_Egger__pval = 0.70 > 0.05) or horizontal pleiotropy (MR‐Egger intercept test: Intercept = −5.82E‐03, *p* = 0.74 > 0.05; MR‐PRESSO global test: RSSobs = 22.55, *p* = 0.75 > 0.05) affecting the MR estimates. The leave‐one‐out analysis confirmed the absence of outlier SNPs (Figure 3C), further strengthening the causal association between depression and CUD.

#### 3.2.3 Assessing the Mediation Proportion of Causal Association

**FIGURE 4:**
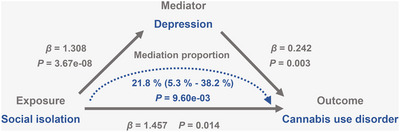
Depression significantly mediates the causal relationship between social isolation and the increased risk of CUD.

The analyses identified three stable causal associations: (1) Increased social isolation significantly elevates the risk of CUD, (2) increased social isolation significantly increases the risk of depression, and (3) increased genetic liability to depression significantly raises the risk of CUD. Based on these findings, it can be inferred that depression may play a crucial mediating role. We evaluated the proportion of mediation using the delta method. As presented in Table 4 and Figure 4, the proportion of mediation attributed to depression was 21.8% (95% CI: 5.3%–38.2%, *Z* = 2.590, *p* = 9.60e‐3). These results highlight the important role of depression in mediating the relationship between social isolation and CUD.

**TABLE 4:** Results of the mediation MR analysis.

Exposure	Mediator	Outcome	Exposure outcome		Exposure mediator		Mediator outcome		Mediation analysis		
			*β* (SE)	*p*	*β* (SE)	*p*	*β* (SE)	*p*	*Z*	*p*	Mediation proportion
Social isolation	Depression	Cannabis use disorder	1.457 (0.590)	0.014	1.308 (0.237)	3.67E‐08	0.242 (0.083)	0.003	2.590	9.60E‐03	21.8 % (5.3%–38.2%)

## 4 Discussion

Using two‐sample MR analysis, this study identified a potential causal effect of social isolation on increased CUD risk. Mediation MR analysis further revealed that depression partially mediated this relationship. The robustness of these findings was supported by multiple supplementary MR methods, which yielded results consistent with the primary IVW method. In addition, various sensitivity analyses further reinforced the reliability and stability of the overall MR results.

Previous observational studies have reported an association between social isolation and an increased risk of CUD. Individuals with CUD have been found to experience lower social support and more negative social interactions (Gulliver and Fowler 2022). Frequent cannabis use among young adults was associated with higher loneliness, greater psychological distress, and lower overall well‐being (Rhew et al. 2021). In recent years, the COVID‐19 pandemic has heightened research attention on the relationship between social isolation, loneliness, and CUD (Bartel et al. 2020). Individuals experiencing moderate or severe loneliness were more likely to increase their cannabis use over time (Gutkind et al. 2022). Older adults in Canada who used cannabis during the COVID‐19 pandemic reported significantly higher levels of loneliness (Li and Deng 2024). Our MR study provides stronger causal evidence on the relationship between social isolation and CUD, building upon previous observational findings.

Previous MR studies have revealed several social and psychological factors influencing the risk of CUD. Lower educational attainment and socioeconomic disadvantage may predispose individuals to higher substance use vulnerability (D. Chen et al. 2023). Meng et al. demonstrated that smoking initiation mediated the causal pathway between sedentary lifestyles and CUD (D. Meng, Wei, et al. 2025). In addition, Ni et al. found that psychological, social, and smoking‐related factors mediated the effects of cannabis use on personality disorders, emphasizing the importance of psychosocial mechanisms (Ni et al. 2025). Taken together, these studies indicate that CUD risk is shaped by complex interactions between social, psychological, and neurobiological factors. To date, no MR study has directly tested social isolation as a causal exposure or evaluated depression as its mediator in relation to CUD. Our findings extend prior evidence by identifying social isolation as a novel social risk factor and quantifying the mediating role of depression.

Our mediation MR analysis demonstrated that depression significantly mediated 21.8% of the causal pathway from social isolation to the risk of CUD. An epidemiological study in the USA found that individuals with mood disorders, including major depressive disorder (MDD), had 3.9 times higher odds of lifetime CUD (Smolkina et al. 2017). Likewise, a Canadian study reported that the 12‐month prevalence of cannabis dependence was seven times higher in those with MDD, while cannabis abuse was 3.5 times more prevalent (Smolkina et al. 2017). Low distress tolerance in depression may increase the risk of CUD by promoting cannabis use as a maladaptive coping mechanism (Buckner et al. 2007). Self‐medication in depression may exacerbate neurotransmitter dysregulation, leading to neuroadaptations in reward circuits and increasing the risk of CUD (Markou et al. 1998). Nevertheless, the estimated mediation proportion should be interpreted with caution, as the analysis was based on summary‐level data. The non‐collapsibility of OR and potential residual confounding between the mediator and outcome may lead to imprecise estimates, and further validation in future studies is warranted (Carter et al. 2021).

These findings have important public health implications. The observed causal relationship between social isolation and CUD underscores the protective role of social connectedness against substance use disorders. Given the increasing prevalence of social isolation, particularly in the wake of the COVID‐19 pandemic, targeted interventions aimed at fostering social engagement could serve as a preventative strategy for reducing CUD risk (O'Sullivan et al. 2021). Community‐based programs, peer support initiatives, and social prescribing—such as linking individuals to social activities like volunteer work or group therapy—may help mitigate the detrimental effects of isolation (Fuller et al. 2022). Furthermore, integrating social support interventions into existing substance use treatment programs may enhance treatment effectiveness and reduce relapse rates.

From a mental health perspective, our findings underscore the need for early identification and intervention for depression in socially isolated individuals. Routine screening for depressive symptoms in individuals experiencing chronic loneliness could facilitate timely mental health interventions, thereby reducing the downstream risk of CUD. Cognitive‐behavioral therapy (CBT) and other psychotherapeutic approaches that enhance emotional regulation and distress tolerance may be particularly beneficial in preventing maladaptive cannabis use among those with depression (Waldron and Kaminer 2004). Overall, our study emphasizes that people with a genetic predisposition to social isolation may particularly benefit from socially oriented preventive measures, which could help translate genetic evidence into practical public health strategies.

The results of this MR study need to be reasonably interpreted. Since SNPs are determined at conception and remain stable throughout life, MR analysis reflects the effect of a lifelong genetic predisposition to social isolation rather than the direct impact of environmental or situational isolation. Furthermore, future research should integrate MR evidence with large‐scale meta‐analyses and randomized controlled trials to comprehensively evaluate causal pathways and intervention potentials (Bartoli et al. 2019; Halicka et al. 2025; Liu et al. 2025; J. Meng et al. 2023, 2024; J. Meng, Li, et al. 2025; Pinto et al. 2019).

This study has several key strengths. First, it is the first to use large‐scale GWAS summary statistics for MR analysis to assess the causal relationship between social isolation and CUD, addressing limitations of previous observational studies with small samples and unclear causality. Second, the consistency of results across multiple supplementary MR methods strengthens the reliability of our findings. Sensitivity analyses further confirm the stability of our results. Third, this study incorporated mediation MR analysis, highlighting depression as a key mediator between social isolation and CUD, offering deeper insight into its mechanisms, and emphasizing the importance of mental health in substance use prevention and intervention. Notably, the GWAS summary statistics for social isolation were derived from questionnaire surveys, which may introduce recall bias. However, the large sample size may mitigate this bias to some extent. Future studies could validate these findings through comprehensive assessments. In addition, this study is based on GWAS summary statistics from European populations; therefore, further validation in other populations is needed in the future. Moreover, the use of summary‐level data precludes assessment of potential exposure‐mediator interactions, which warrants further investigation in future studies.

## 5 Conclusion

This study demonstrates that social isolation is significantly associated with an increased risk of CUD, with depression playing a key mediating role in this causal relationship. These findings highlight the importance of addressing social isolation and implementing targeted mental health interventions to reduce depression, thereby mitigating CUD risk. Nonetheless, the results should be interpreted with caution due to potential recall bias, the focus on European ancestry samples, and the limited capacity of summary‐level data to assess exposure‐mediator interactions or residual confounding.

## Author Contributions

**Tao Ma**: conceptualization, methodology, formal analysis, investigation, data curation, writing – original draft, visualization, validation, software, project administration, resources.

## Funding

This research was supported by the Science and Technology Plan Project of the Ministry of Public Security (Grant No. 2023YY21) and General Project of the Hunan Provincial Social Science Fund (Grant No. 24YBA097).

## Ethics Statement

The GWAS summary statistics used in this study were all obtained from public databases, and the corresponding original studies had received ethical approval. Therefore, no additional ethical approval or informed consent was required for this study.

## Consent

The author has nothing to report.

## Conflicts of Interest

The author declares no conflicts of interest.

## Supporting information




**Supplementary Tables**: brb371102‐sup‐0001‐TableS1‐S9.xlsx

## Data Availability

Supplementary Table S1 presents the details of the GWAS summary statistics for MR analysis.
